# Increased expression of the ubiquitin – proteasome pathway in murine myotubes by proteolysis-inducing factor (PIF) is associated with activation of the transcription factor NF-*κ*B

**DOI:** 10.1038/sj.bjc.6601132

**Published:** 2003-09-09

**Authors:** A S Whitehouse, M J Tisdale

**Affiliations:** 1 Pharmaceutical Sciences Research Institute, Aston University, Birmingham B4 7ET, UK

**Keywords:** cachexia, protein catabolism, proteolysis-inducing factor, EPA, NF-*κ*B, proteasome expression

## Abstract

Proteolysis-inducing factor (PIF), isolated from a cachexia-inducing murine tumour, has been shown to stimulate protein breakdown in C_2_C_12_ myotubes. The effect was attenuated by the specific proteasome inhibitor lactacystin and there was an elevation of proteasome ‘chymotrypsin-like’ enzyme activity and expression of 20S proteasome *α*-subunits at concentrations of PIF between 2 and 16 nM. Higher concentrations of PIF had no effect. The action of PIF was attenuated by eicosapentaenoic acid (EPA) (50 *μ*M). At a concentration of 4 nM, PIF induced a transient decrease in I*κ*B*α* levels after 30 min incubation, while no effect was seen at 20 nM PIF. The level of I*κ*B*α*, an NF-*κ*B inhibitory protein, returned to normal after 60 min. Depletion of I*κ*B*α* from the cytosol was not seen in myotubes pretreated with EPA, suggesting that the NF-*κ*B/I*κ*B complex was stabilised. At concentrations between 2 and 8 nM, PIF stimulated an increased nuclear migration of NF-*κ*B, which was not seen in myotubes pretreated with EPA. The PIF-induced increase in chymotrypsin-like enzyme activity was also attenuated by the NF-*κ*B inhibitor peptide SN50, suggesting that NF-*κ*B may be involved in the PIF-induced increase in proteasome expression. The results further suggest that EPA may attenuate protein degradation induced by PIF, at least partly, by preventing NF-*κ*B accumulation in the nucleus.

Loss of protein from skeletal muscle is a common phenomenon associated with a number of catabolic conditions. Of the proteolytic pathways, the lysosomal (cathepsins) and the calcium-dependent cysteine proteases (calpains) contribute between 15 and 20% of total protein breakdown in muscles (Lecker *et al*, 1999). Muscle wasting in starvation (Wing and Goldberg, 1993), sepsis (Tiao *et al*, 1994), metabolic acidosis (Mitch *et al*, 1994), weightlessness (Taillandier *et al*, 1996), severe trauma (Biolo *et al*, 2000), denervation atrophy (Medina *et al*, 1995) and cancer cachexia, in both mice (Lorite *et al*, 1998) and humans (Williams *et al*, 1999) has been attributed to upregulation of ATP–ubiquitin-dependent proteolysis (ubiquitin–proteasome). In this process, proteins are tagged for degradation by the attachment of a polyubiquitin chain, which is recognised by the 26S proteasome, a large multisubunit catalytic complex. However, the ubiquitin–proteasome pathway does not disassemble myofibrils directly and the calcium–calpain pathway has been suggested to release myofilaments from the sarcomere before myosin can be degraded by the proteasome (Hasselgren and Fischer, 2001).

Muscle protein degradation in cancer cachexia appears to be associated with a sulphated glycoprotein of Mr 24 000 secreted by cachexia-inducing murine and human tumours (Todorov *et al*, 1996a; Cabal-Manzano *et al*, 2001). This substance is capable of inducing muscle protein breakdown directly, both *in vitro* and *in vivo* (Lorite *et al*, 1997), and has been named proteolysis-inducing factor (PIF). PIF has been detected in the urine of weight-losing patients with carcinoma of the pancreas, breast, ovary, lung, colon, rectum and liver (Cariuk *et al*, 1997). Patients with pancreatic cancer excreting PIF in the urine had a significantly greater total weight loss and rate of weight loss than patients whose urine did not contain PIF (Wigmore *et al*, 2000). Protein degradation induced by PIF, both in gastrocnemius muscle *in vivo* and murine myoblasts *in vitro*, is associated with increased levels of both mRNA and protein for the Mr 14 000 ubiquitin conjugating protein and proteasome *α* and *β* subunits (Lorite *et al*, 2001), suggesting that the ubiquitin-proteasome proteolytic pathway plays a major role in the action of PIF. The induction of protein degradation by PIF has been shown to be completely attenuated by pretreatment with eicosapentaenoic acid (EPA) (Lorite *et al*, 1997), which has been shown to downregulate expression of the key regulatory components of the ubiquitin–proteasome pathway in skeletal muscle of mice bearing the MAC16 tumour (Whitehouse *et al*, 2001a).

Although PIF has been shown to upregulate expression of the ubiquitin–proteasome proteolytic pathway in murine myotubes (Gomes-Marcondes *et al*, 2002), there is no information, to date, on nuclear transcription factors involved. In primary hepatocytes and the human cancer cell line Hep G2, PIF has been shown to activate the transcription factor nuclear factor-kappa B (NF-*κ*B) resulting in the increased production of interleukin-8 (IL-8), interleukin-6 (IL-6) and C-reactive protein, and the decreased production of transferrin (Watchorn *et al*, 2001). There was also an increase in ICAM-1, another NF-*κ*B inducible gene. Protein degradation induced by TNF-*α* in murine myotubes also appears to arise from an increase in ubiquitin-dependent proteolysis (Li *et al*, 1998) and studies in transfected cells containing mutant I*κ*B*α* proteins insensitive to degradation suggested that NF-*κ*B was an essential mediator of the TNF-*α*-induced protein breakdown (Li and Read, 2000). This suggests that the induction of protein degradation by PIF may also be mediated through NF-*κ*B. The aim of the present study was to investigate the effect of PIF on the activation of NF-*κ*B in C_2_C_12_ murine myotubes at concentrations causing an increased expression of the ubiquitin–proteasome pathway. Since EPA has been shown to attenuate the catabolic effect of PIF in murine myoblasts (Smith *et al*, 1999) the effect of this essential fatty acid on protein catabolism, activation of the ubiquitin–proteasome pathway and nuclear accumulation of NF-*κ*B by PIF has been studied in myotubes to determine potential mechanisms of signal transduction.

## METHODS

### Purification of PIF

Pure strain female NMRI mice were obtained from our own breeding colony and were transplanted with fragments of the MAC 16 tumour into the flank by means of a trocar (Bibby *et al*, 1987). MAC16 tumours were originally derived from colon tumours induced by dimethylhydrazine in NMRI mice (Cowen *et al*, 1980). PIF was isolated from tumours that were excised, once weight loss was established (20–25%). All animal experiments have been carried out with approval from the British Home Office and the ethical guidelines followed meet the standards required by the UKCCR guidelines (Workman *et al*, 1998). The tumours were homogenised in 5 ml g^−1^ of a Tris buffer (Tris 3.03 g, EGTA 0.4755 g, DTT, 0.3865 g: (PMSF 0.1 M in 12.5 ml isopropanol in 2500 ml). All reagents were obtained from Sigma-Aldrich, Dorset, UK unless otherwise stated. After centrifugation (3100 **g**, 15 min), 38% w v^−1^ ammonium sulphate was slowly added to the supernatant while stirring on ice. The solution was then left stirring overnight at 4°C. Centrifugation at 4000 **g** for 20 min was followed by ultracentrifugation at 20 000 **g** for 35 min to remove any fat, after which time the homogenate was dialysed several times in a 10 000 MW cutoff ultrafiltration cell against two to three changes of 300 ml PBS. The retenate was centrifuged at 4000 **g** for 20 min and the sample was circulated on an affinity column overnight. The column itself contains antibodies raised against murine PIF (Todorov *et al*, 1996b), which are coupled to protein A on an Affi-Gel column. PIF was eluted from the column the next day with 0.1 M glycine, pH 2.5, into tubes containing 500 *μ*l Tris pH 8.0. It was then redialysed in an equal volume PBS and concentrated to 0.5–1.0 ml. The purity of PIF was confirmed by polyacrylamide gel electrophoresis and immunoblotting. This showed a band for PIF at Mr 24 000, sometimes accompanied by an albumin bound band at Mr 69 000 (Todorov *et al*, 1996b). No other bands were apparent.

### Cell culture and differentiation of C_2_C_12_ myoblasts into myotubes

All experiments were performed with myotubes since they contain the myofibrillar proteins actin and myosin seen in skeletal muscle. C_2_C_12_ myoblasts were maintained in a proliferative state of growth in Dulbecco's modified Eagle's medium (DMEM) supplemented with 5% foetal calf serum and 1% penicillin/streptomycin (Gibco Life Sciences, UK). For all experimentation, cells were differentiated into myotubes by incubation in DMEM, which had been supplemented with 2% horse serum and 1% penicillin/streptomycin when the myoblasts became confluent. The medium was changed every other day and differentiation occurred in 5–7 days.

### Measurement of protein degradation

Protein degradation was determined as previously described (Gomes-Marcondes *et al*, 2002) in six well multiwell dishes. Just before the cells were fully differentiated, 20 *μ*l of [^3^H] phenylalanine ([^3^H]-Phe) corresponding to 2 *μ*Ci was added to each well and the cells were incubated at 37°C in 5% CO_2_ overnight in the absence of any treatment. [^3^H]-Phe was preprepared by adding 60 mg ‘cold’ phenylalanine to 500 *μ*l [^3^H]-Phe in 4500 *μ*l PBS. The following day (during which time myotubes would have fully formed), the media was discarded and the cells were rinsed twice in PBS. DMEM without phenol red (supplemented with 2% horse serum and 1% penicillin/streptomycin) was added following a 2 h preincubation, the media was removed and replaced with DMEM without phenol red (supplemented as previous) along with a range of concentrations of PIF with or without lactacystin (10 *μ*M) and 2 mM ‘cold’ phenylalanine. Cells were then incubated for a further 24 h. Finally, 1 ml of supernatant was removed and added to 6 ml optiphase ‘Hi safe 3’ scintillation fluid (Fisher Chemicals, Leicester, UK). The radioactivity released was analysed using a ‘Tri-carb 2000CA’ liquid scintillation analyser (United Technologies, Packard, Berks, UK). The data were the same whether expressed as total [^3^H]-Phe released or as a percentage of total radioactivity incorporated.

### Measurement of proteasomal ‘chymotrypsin-like’ enzyme activity in C_2_C_12_ myotubes

The ‘chymotrypsin-like enzyme activity of the proteasome was determined fluorometrically according to the method of Orino *et al* (1991). Myotubes were pretreated with EPA for 2 h prior to the addition of PIF and the EPA remained in the culture medium for the course of the experiment. After 24 h, myotubes were washed twice in ice-cold PBS and scraped into approximately 0.5 ml homogenising buffer (20 mM Tris, pH7.5, 2 mM ATP, 5 mM MgCl_2_, 50 mM DTT). Samples were then sonicated for three pulses of 15 s, with 10 s intervals, with care to avoid heating, and centrifuged at 18 000 **g** for 10 min at 4°C to pellet insoluble material. A stock solution of substrate (10 mg *N*-succinyl-LLVY-7-amido-4-methyl coumarin in 600 *μ*l dimethyl sulphoxide) was diluted 1 : 1000 in 100 mM Tris/HCI, pH 8.0, for use. A volume of 100 *μ*l was added to 50–100 *μ*l of prepared sample. A duplicate set of samples was included to which the proteasome inhibitor lactacystin (Affiniti Research Products, Exeter, Devon, UK) had been added to give a final concentration of 10 *μ*M per well. Samples were then incubated for 2 h on ice. Fluorescence of the substrate was measured using an LS50 Luminescence Spectrometer (Perkin-Elmer) at excitation 360 nm and emission 460 nm, and values were adjusted for equal protein concentrations and for a reaction blank. The substrate concentration was saturated and the activity was linear with protein concentration.

### Determination of protein concentration

Protein concentrations were determined using a standard commercially available colourimetric protein assay (Biorad, UK) according to the manufacturer's instructions.

#### Western blotting protocol

Myoblasts were allowed to differentiate into myotubes as above, and treated with various concentrations of PIF as indicated in the figure legends, with or without 2 h preincubation with 50 *μ*M EPA. The media were rinsed from myotubes that were homogenised and sonicated in 500–2000 *μ*l homogenising buffer (as described above). After centrifugation (18 000 **g** for 5 min) to pellet-insoluble material, the supernatant was assayed for protein concentration. Homogenates were denatured in electrophoresis sample buffer (125 mM Tris, pH 6.8, 4% SDS, 10% glycerol, 0.006% bromophenol blue, 2% mercaptoethanol) by heating to 95°C for 5 min and electrophoresed on a 12% SDS–polyacrylamide gel (10 cm × 10 cm) along with ‘Rainbow’ molecular weight markers (Amersham Biosciences, UK).

Proteins were electrotransferred to a nitrocellulose membrane using an enclosed system for 2 h at a constant voltage of 80 V. After transfer, the membranes were rinsed in wash buffer (0.1% Tween 20 in PBS) and transferred to blocking buffer (5% Marvel in wash buffer) for 1 h at room temperature. Blots were probed for 20S proteasome *α*-subunits, p42 proteasome subunit and inhibitory protein of NF-*κ*B (I*κ*B*α*) using appropriate antisera. Primary antisera were diluted in blocking buffer (Rabbit polyclonal I*κ*B*α*1 : 400 Autogen Bioclear, UK anti-p42 1 : 120 and mouse monoclonal 20S*α* 1 : 1500 Affiniti Research Products, Exeter, Devon, UK) and added to membranes for 1 h at room temperature. This was followed by washing with 0.1% PBS-Tween for 1 h at room temperature with agitation, changing the wash buffer every 15–20 min. Anti-mouse or anti-rabbit IgG : HRP (horseradish peroxidase), diluted 1 : 2000 in wash buffer, was added and incubated for 1 h at room temperature. After incubation with secondary antibody, the membranes were washed for a further 90 min with agitation, again changing the buffer every 15–20 min.

Proteins were detected using an ‘Enhanced Chemiluminescence’ (ECL) system (Amersham Biosciences, UK), which is based upon the oxidation of luminol by HRP, resulting in light emission, detected by a blue light sensitive autoradiography film. To ensure equal loading, a parallel gel was stained with 1% Coomassie Brilliant Blue (in 40% methanol/10% acetic acid) for 1 h.

#### Electrophoretic mobility shift assay (EMSA)

**Figure 5:**
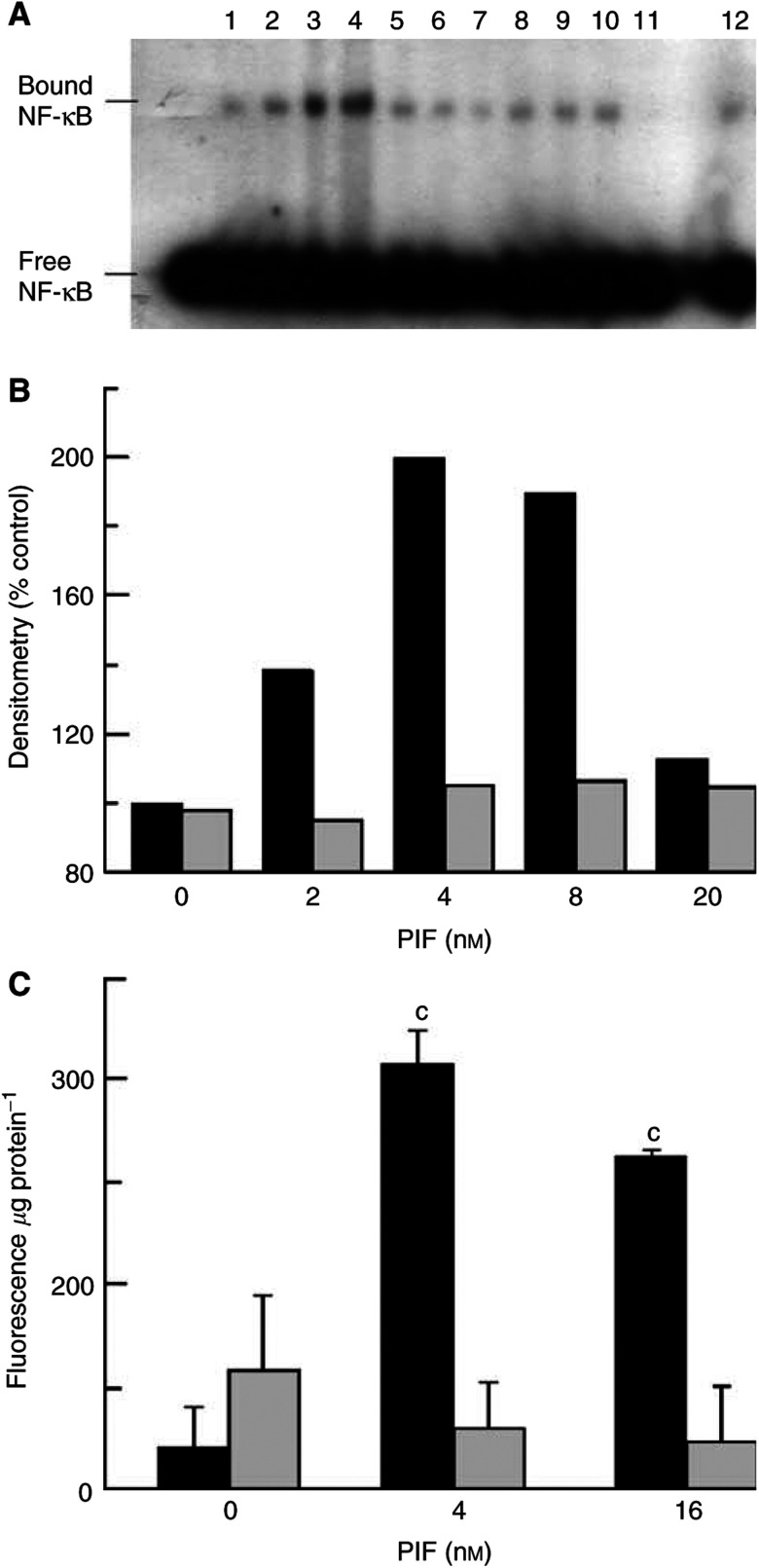
(**A**) Effect of PIF on the electrophoretic mobility of (*γ*^32^P) NF*κ*B in C_2_C_12_ myotubes in the absence (lanes 1–5) and presence (lanes 6–10) of 50 *μ*M EPA. The following additions were made: lanes 1 and 60 nM PIF; lanes 2 and 7, 4 nM PIF; lanes 3 and 8, 8 nM PIF, lanes 4 and 9, 16 nM PIF and lanes 5 and 10, 20 nM PIF. Lane 11 contains a competitor, that is an equal concentration of unlabelled NF-*κ*B, while lane 12 contains a noncompetitor, an equal concentration of an unlabelled, unrelated oligonucleotide, usually AP2. (**B**) Densitometric analysis of the blot shown in (**A**) Values shown are in the absence (black boxes) or presence (hatched boxes) of 50 *μ*M EPA. The blot shown is representative of three separate blots performed on different occasions. (**C**) The effect of SN50 on PIF-induced upregulation of the chymotrypsin-like activity of the proteasome measured after 24 h incubation. Solid boxes indicate PIF alone, hatched boxes indicate PIF+18 *μ*M SN50. The SN50 was added 2 h prior to PIF and remained in the culture medium throughout the experiment. Sample size per treatment group *n*=9 and the experiment was repeated twice. Differences from control and SN50 treated cells are shown as c, *P*<0.001.

C_2_C_12_ myotubes were treated with EPA and PIF as described in Figure 5A

. A vehicle control (as well as no treatment control) was included, to differentiate any effects caused by the solvent. The cells were incubated for 20 min with PIF prior to the EMSA assay.

#### Labelling of consensus oligonucleotides

In all, 2 *μ*l NF-*κ*B (1.75 pmol *μ*l^−1^) (Promega UK), oligonucleotide sequence 5′-AGT TGA GGG GAC TTT CCC AGG C-3′; 3′-TCA ACT CCC CTG AAA GGG TCC G-5′; 1 *μ*l T4 polynucleotide kinase 10 × buffer (Promega, UK); 2 *μ*Ci [*γ*-^32^P]ATP (Amersham Biosciences, UK); 1 *μ*l T4 polynucleotide kinase (Promega, UK) were assembled in a sterile microcentrifuge tube, the volume was adjusted to 10 *μ*l with nuclease-free H_2_O (Promega, UK), and incubated at 37°C for 1 h. The reaction was stopped by the addition of 2 *μ*l 0.5 M EDTA followed by 88 *μ*l of TE buffer.

#### Preparation of nuclear proteins

The cells were rinsed, scraped and pelleted in wash buffer (10 mM HEPES/KOH pH 7.5, 10 mM KCl, 2 mM MgCl_2_, 1 mM DTT, 0.1 mM EDTA, 0.4 mM PMSF, 0.2 mM NaF, 0.2 mM sodium orthovanadate, 0.3 mg ml^−1^ leupeptin). Extracts were prepared according to Johnson *et al*. (1996) with modifications. The pellets were resuspended in 300 *μ*l of the same wash buffer and incubated on ice for 15 min. In all, 30 *μ*l of 1% ‘Triton X-100’ (octylphenoxy-polyethoxyethanol) was then added and the cells were lysed by vortexing. A 4-min centrifugation at 2000 **g** pelleted the nuclei and the supernatant was removed. The nuclear pellet was resuspended in 50 *μ*l of the ice-cold high-salt buffer (50 mM HEPES, pH 7.8, 50 mM KCl, 300 mM NaCl, 0.1 mM EDTA, 1 mM DTT, 0.4 mM PMSF, 0.2 mM NaF, 0.2 mM sodium orthovanadate, 10% glycerol) to solubilise nuclear proteins and the suspension was kept on ice for 20 min with a 30 s vortex every 3–5 min. A centrifugation at 15 500 **g** for 3 min yielded the supernatant containing the protein extract. The concentration of nuclear extracts was measured by protein assay (as described previously). Measurements were repeated three times to ensure the accuracy of the assay.

#### DNA binding reaction

Gel shift binding buffer (2 *μ*l) (Promega, UK) and nuclear extract (10 *μ*g for each test) were added in a sterile microfuge tube, along with 2 *μ*l unlabelled NF*κ*B (competitor control) or 2 *μ*l of a different unlabelled oligonucleotide (noncompetitor control). A negative control reaction was also included, which contained gel shift binding buffer and no sample. In total, 2 *μ*l of [*γ*-^32^P] NF*κ*B was then added to tests and controls and the volumes were equalised with nuclease-free water. The reactions were then incubated at room temperature for 2 h. Finally, 1 *μ*l of gel loading 10 × buffer (250 mM Tris-HCI, pH 7.5, 0.2% bromophenol blue, 40% glycerol) was added to the negative control, and the reaction products were analysed via electrophoresis on an 8% nondenaturing polyacrylamide gel, with a native 5% polyacrylamide stacking gel, which had been allowed to polymerise for at least 2 h. The gels were pre-electrophoresed for 10 min at 150 mV and then electrophoresed (after the addition of samples) at 150 mV for approximately 30 min, or until the bromophenol blue dye front reached the base of the gel. The gel was then dried between Whatman 3 MM filter paper and plastic wrap and exposed to ‘Hyperfilm MP’ for 8–16 h at −70°C.

### Statistical analysis

Data are expressed as mean±s.e.m. and each experiment was repeated at least three times. Differences were analysed by two-way ANOVA followed by Tukey's post-test. Differences were considered significant if *P*<0.05.

## RESULTS

**Figure 1:**
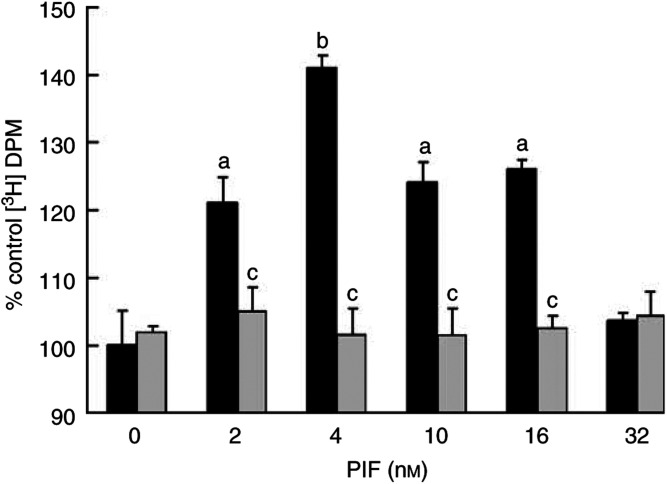
Effect of PIF in the absence (black boxes) or presence (grey boxes) of lactacystin (10 *μ*M) on protein degradation in C_2_C_12_ myotubes as determined by [^3^H] phenylalanine release. Lactacystin was present for 2 h prior to PIF and was also present throughout the 24 h incubation period. All values have been normalised to their respective controls and the experiment was repeated twice, *n*=3 for each treatment. The data are expressed as mean±s.e.m. and was measured 24 h after the addition of PIF. Differences from control are indicated as a, *P*<0.01 and b, *P*<0.001, while differences in the presence of lactacystin are shown as c, *P*<0.001.

**Figure 2:**
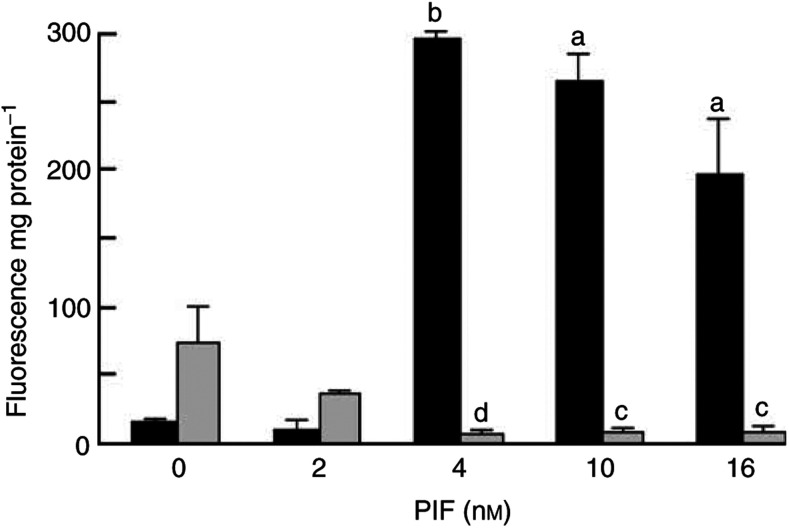
Effect of PIF on the chymotrypsin-like enzyme activity in C_2_C_12_ myotubes in the absence (black boxes) or presence (grey boxes) of 50 *μ*M EPA (grey boxes). The data are expressed as mean±s.e.m. where *n*=9 and the values represent the activity inhibited by 10 *μ*M lactacystin. The differences from control are indicated as a, *P*<0.05 and b, *P*<0.001, while differences in the presence of EPA are indicated as c, *P*<0.05 and d, *P*<0.001.

**Figure 3:**
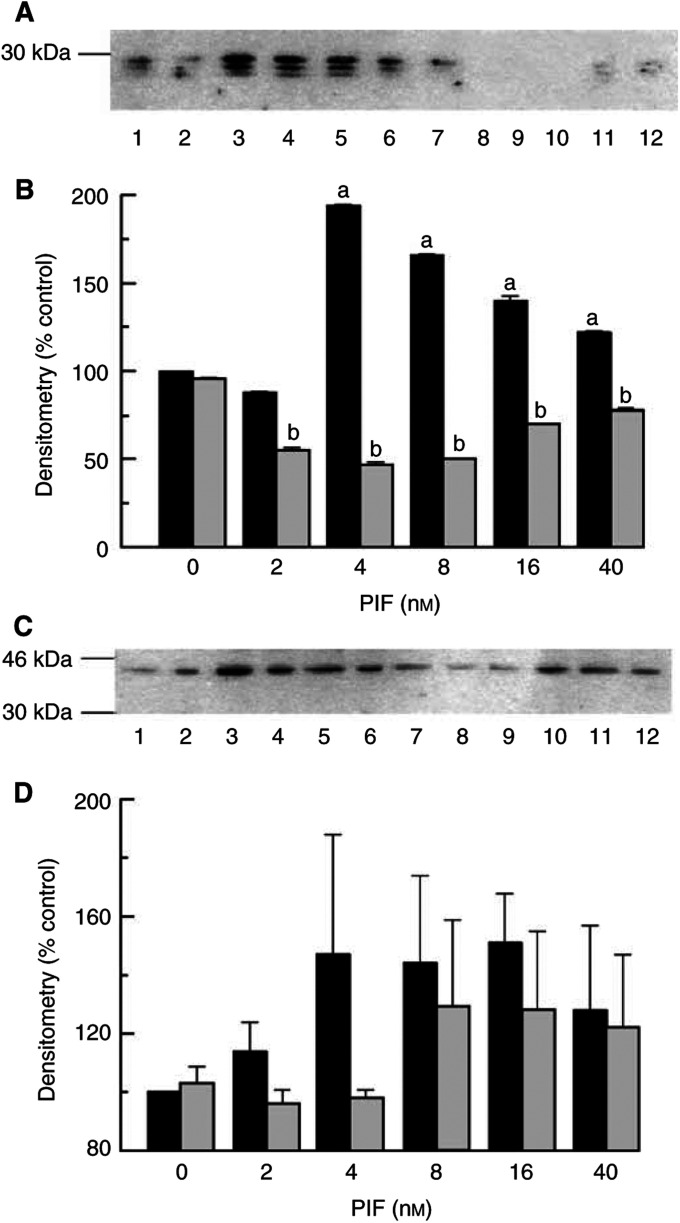
Western blot analysis of 20S proteasome *α*-subunit expression (**A**) or (**C**) the 19S subunit, p42 in C_2_C_12_ myotubes 24 h after addition of PIF alone at 0 nM (lane 1), 2 nM (lane 2), 4 nM (lane 3), 8 nM (lane 4), 16 nM (lane 5) or 40 nM (lane 6) or after pretreatment with 50 *μ*M EPA for 2 h and subsequent treatment with PIF at 0 nM (lane 7), 2 nM (lane 8), 4 nM (lane 9), 8 nM (lane 10), 16 nM (lane 11) and 40 nM (lane 12). (**B**) Densitometric analysis of the blot shown in (**A**), *n*=2. (**D**) Densitometric analysis of the blot shown in (**C**), *n*=2. Values shown are in the absence (black boxes) or presence (stippled boxes) of 50 *μ*M EPA. Differences from control are shown as a, *P*<0.001, while differences from PIF-treated cells are shown as b, *P*<0.001.

In the current experiments, myotubes were used as a model of skeletal muscle, since they contain the myofibrillar proteins actin and myosin not found in myoblasts. When C_2_C_12_ murine myotubes labelled with [^3^H] phenylalanine were treated with PIF for 24 h, there was an increase in total protein degradation, as evidenced by increased release of radiolabel (Figure 1

). The maximal protein breakdown was seen at concentrations of PIF between 2 and 16 nM, with a maximal peak of stimulatory activity (41% increase over control) at 4 nM PIF. These concentrations are in the range expected from maximal tumour release into the circulation (35 nM) (Todorov *et al*, 1996a). Protein degradation was attenuated in the presence of the highly specific and irreversible proteasome inhibitor lactacystin, which does not inhibit lysosomal protein degradation (Fenteany and Schreiber, 1998) at a concentration of 10 *μ*M (Figure 1), indicating that the elevated catabolism was due to increased proteasome activity. There was no effect of lactacystin on cell viability. Pilot experiments suggested that this concentration was sufficient to attenuate proteasome activity. PIF induced an increase in the chymotrypsin-like activity of the proteasome (the dominant catalytic activity of the *β*-subunits), with an increase of more than 10-fold in the presence of 4 nM PIF (Figure 2

). The effect was completely abolished in the presence of lactacystin, confirming the specificity of the response, and the data presented in Figure 2 represent the lactacystin-suppressible activity. The PIF-induced increase in chymotrypsin-like activity of the proteasome was also attenuated by 50 *μ*M EPA (Figure 2), which has also been shown to attenuate protein catabolism induced by PIF in C_2_C_12_ myoblasts (Smith *et al*, 1999). Lower concentrations of EPA (25 *μ*M) were ineffective. The plasma concentration of EPA in humans consuming therapeutic levels of this fatty acid might be expected to be in the range 100–200 *μ*M. As with protein degradation, chymotrypsin-like activity exhibited a bell-shaped dose–response curve. A similar effect was observed for expression of 20S proteasome subunits as detected by Western blotting (Figure 3A

) as well as for p42, an ATPase subunit of the 19S regulator (Figure 3C). After 24 h incubation with PIF, maximal increase in expression was seen between 4 and 16 nM PIF with an almost two-fold increase at 4 nM PIF (Figure 3B and D). Myotubes treated with 50 *μ*M EPA for 2 h prior to the addition of PIF showed no increase in the expression of 20S *α*-subunits at any PIF concentration (Figure 3), although there was no effect on expression of p42 at the higher concentrations of PIF. These results confirm the ability of EPA to attenuate the increased expression of 20S proteasome subunits induced by PIF.

**Figure 4:**
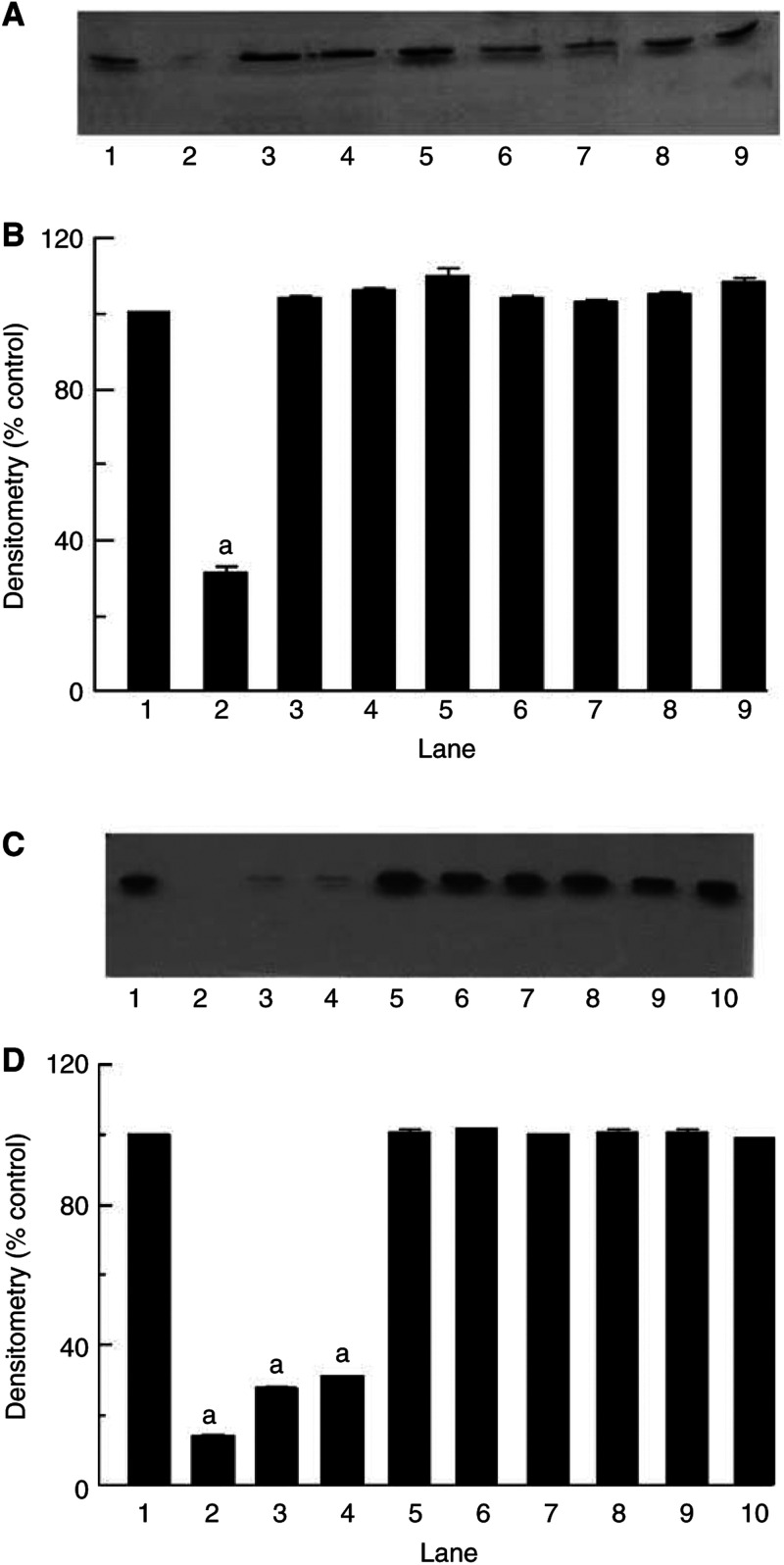
(**A**) The effect of PIF on I*κ*B*α* expression in C_2_C_12_ myotubes at 30, 60 and 120 min as determined by Western blotting. Lanes 1, 2 and 3 represent 0, 4 and 20 nM PIF after 30 min; lanes 4–6 after 60 min and lanes 7–9 after 120 min. (**B**) Densitometric analysis of the blot shown in (**A**) *n*=2. Differences from control are shown as a, *P*<0.001. (**C**) The effect of PIF on I*κ*B*α* expression in C_2_C_12_ myotubes after 30 min in the absence (lanes 1–5) or presence (lanes 6–10) of 50 *μ*M EPA added 2 h prior to PIF. The lanes represent the following concentrations of PIF: 1 and 6, 0 nM; 2 and 7, 2 nM; 3 and 8, 4 nM; 4 and 9, 8 nM and 5 and 10, 20 nM. (**D**) Densitometric analysis of the blot shown in (**C**), *n*=2. Differences from control are shown as a, *P*<0.001.

A time course for the effect of PIF on I*κ*B*α* levels in C_2_C_12_ myotubes is shown in Figure 4A

. There was a transient decrease in cytosolic I*κ*B*α* levels after 30 min incubation with PIF at 4 nM, but not at 40 nM. When analysed densitometrically, the level of I*κ*B*α* was found to decrease by 75% from the control value within 30 min of the addition of 4 nM PIF (Figure 4B). The level of I*κ*B*α* returned to normal at 60 min and remained at this level indicating that the cell had recovered. When treated with a wider concentration range of PIF there was a decrease in I*κ*B*α* levels in all myotubes treated with all concentrations of PIF inducing protein degradation (Figure 4C). Thus, after 30 min there was a decrease of 99.9% at 2 nM PIF, 89.7% at 4 nM PIF and 87.1% at 8 nM PIF, as determined by densitometric analysis, but no change at 20 nM PIF (Figure 4D). Depletion of I*κ*B from the cytosol was not seen in myotubes pretreated with EPA, suggesting an effect of EPA in stabilising the NF*κ*B/I*κ*B complex in the cytoplasm.

The effect of PIF on NF*κ*B activation and accumulation in the nucleus was determined by EMSA. Proteolysis-inducing factor stimulated an increased nuclear migration of NF*κ*B at concentrations between 2 and 8 nM (Figure 5A and B) but not at higher concentrations. Only one sequence specific shift was identified by competition assays, which may suggest stimulation of a single dimer pair by PIF. The specificity of the band to NF-*κ*B was confirmed from preliminary experiments using HeLa nuclear extracts and myotubes exposed to endotoxin. The increased nuclear levels of NF*κ*B were not seen when PIF was added to myotubes pretreated with 50 *μ*M EPA (Figure 5A). The increased levels of NF*κ*B demonstrated in the nucleus in response to PIF and the attenuation of this by EPA correspond to those concentrations which induce both degradation of I*κ*B*α* and increased proteasome activity. The NF*κ*B inhibitor peptide, SN50, also attenuated the PIF-induced increase in chymotrypsin-like-enzyme activity (Figure 5B) and reduced nuclear NF-*κ*B in the presence of 4 nM PIF by 80%. This suggests that NF*κ*B may be involved in the PIF-induced increase in proteasome expression.

## DISCUSSION

*In vivo* studies in mice bearing a cachexia-inducing colon adenocarcinoma (MAC16) have shown EPA to attenuate effectively the development of host weight loss in a dose-dependent manner, with preservation of both adipose tissue and skeletal muscle mass (Beck *et al*, 1991). EPA has also been shown to stabilise body weight in weight-losing patients with advanced pancreatic cancer (Wigmore *et al*, 2000). Animals bearing the MAC16 tumour showed a decreased protein synthesis and an increased protein degradation in skeletal muscle, and treatment with EPA significantly reduced protein degradation without an effect on protein synthesis (Beck *et al*, 1991). This effect has been shown to occur by downregulation of the increased expression of the regulatory components of the ubiquitin–proteasome proteolytic pathway seen in skeletal muscle of cachectic mice (Whitehouse *et al*, 2001a). This study provides some evidence for the mechanism by which this occurs.

Previous studies (Lorite *et al*, 2001; Gomes-Marcondes *et al*, 2002) have shown that PIF induces protein degradation in skeletal muscle by upregulation of the ubiquitin–proteasome proteolytic pathway. This study shows that the effect is concentration dependent with only those concentrations between 2 and 16 nM inducing protein breakdown. These same concentrations of PIF increased expression of the 20S proteasome *α*-subunits and also increased proteasome proteolytic activity, as determined by the chymotrypsin-like enzyme activity, the predominant proteolytic enzyme activity of the *β*-subunits. Protein degradation and activation of the ubiquitin–proteasome pathway by PIF was attenuated by lactacystin, showing the specificity of the response and also by EPA, as occurs in the gastrocnemius muscle of mice bearing the cachexia-inducing MAC16 tumour (Whitehouse *et al*, 2001a). In contrast, the related fatty acid docosahexaenoic acid (DHA) has been shown to be ineffective in attenuating protein catabolism and increased expression of the ubiquitin–proteasome pathway (Whitehouse *et al*, 2001b). This shows that PIF induces proteasome expression in myotubes through an intracellular signalling cascade that is sensitive to EPA. Previous studies (Smith *et al*, 1999) suggested that 15-hydroxyeicosatetraenoic acid (15-HETE) acted as a signal for protein degradation induced by PIF and that formation of 15-HETE was sensitive to EPA. The present study provides some evidence for a role of NF-*κ*B in skeletal muscle proteolysis induced by PIF and other studies (unpublished results) have also shown 15-HETE to induce increased expression of the ubiquitin–proteasome pathway through an NF-*κ*B-mediated process.

NF-*κ*B is accepted to regulate the transcription of genes involved in immune responses, cell growth and cell death (Baeurele and Baltimore, 1996). While in most cell types, NF-*κ*B seems to promote cell proliferation and protect against apoptosis, in skin it appears to oppose proliferation (Dajee *et al*, 2003). Thus, NF-*κ*B can apparently have different functions in different cell types. The role of proteolysis in skeletal muscle and the relationship to expression of proteasome subunits and other key steps in the ubiquitin–proteasome pathway are also confusing. Thus, Du *et al* (2000) demonstrated that NF-*κ*B was a repressor of C3 proteasome subunit expression in rat L6 muscle cells. It has been previously shown (Grune *et al*, 1996) that preventing the transcription of just one subunit (C2) reduced not only the number of functioning proteasomes, but also proteolytic activity and protein degradation. Glucocorticoids were shown to stimulate C3 subunit expression by opposing the suppressor action of NF-*κ*B by antagonising the interaction of the NF-*κ*B protein with an NF-*κ*B response element in the C3 subunit promoter region. L6 myotubes that had been heat shocked were protected from the catabolic effects of dexamethasone, together with attenuation of the downregulation of NF-*κ*B (Luo *et al*, 2001). In sepsis, there is an early (4 h) upregulation of NF-*κ*B activity in skeletal muscle, followed by inhibited NF-*κ*B at 16 h (Penner *et al*, 2001). The latter effect was probably due to glucocorticoids since the glucocorticoid receptor antagonist RU38486 increased NF-*κ*B. Incubation of L6 cells with a mixture of cytokines, including TNF*α*, decreased C3 proteasome subunit expression and increased the amount of activated NF-*κ*B.

However, other studies suggest a positive correlation between activation of NF-*κ*B and expression of genes of the ubiquitin–proteasome proteolytic pathway. Li *et al* (1998) showed that differentiated murine myotubes treated with tumour necrosis factor *α* (TNF*α*) lost total protein and adult myosin heavy chain, together with activation of NF-*κ*B. They showed that the activity of NF-*κ*B could be correlated to an increased ubiquitin conjugation to muscle proteins, and a subsequent rise in ubiquitin mRNA, suggesting that NF-*κ*B might function to increase expression of members of the ubiquitin–proteasome pathway in skeletal muscle. This conclusion was endorsed by studies using C_2_C_12_ myotubes transfected with viral plasmid constructs that overexpress mutant I*κ*B*α* proteins that are insensitive to degradation by the ubiquitin–proteasome pathway (Li and Read, 2000). Total protein content and myosin heavy-chain levels were found to be unaltered in response to TNF*α*, suggesting that NF-*κ*B is an essential mediator of TNF*α*-induced protein catabolism in differentiated muscle cells. It is possible that this apparent disparity among the literature may reflect cell-system specific recruitment of NF-*κ*B subunits, which may have different biological effects. Indeed, it has already been suggested that NF-*κ*B may be an intermediate in both catabolic and anabolic pathways (Mitch and Price, 2001).

The present study provides some support to the work of Li and Read (2000) that NF-*κ*B provides a positive signal for protein degradation in skeletal muscle and may act to increase expression of proteasome subunits and ubiquitin-dependent proteolysis in murine myotubes. Certainly, nuclear accumulation of NF-*κ*B is increased by PIF at the same concentrations that induce protein catabolism and upregulation of 20S proteasome *α*-subunit expression and this is accompanied by the disappearance of I*κ*B*α* from the cytosol. In addition, the NF-*κ*B inhibitor peptide, SN50, which competes for the nuclear translocation sequences of NF-*κ*B, attenuated both the increased DNA binding of NF-*κ*B induced by PIF and the increased proteasome chymotrypsin-like enzyme activity. Both proteasome expression and nuclear accumulation of NF-*κ*B induced by PIF were also attenuated by EPA, which acted to prevent the degradation of I*κ*B*α*, possibly by stabilising the NF-*κ*B/I*κ*B complex directly. EPA may exert its effects upon the inhibition of I*κ*B*α* degradation through interference with upstream effectors, like I*κ*B kinase kinase (IKK), possibly by direct binding via a thioester or O-ester linkage as occurs in platelets (Muszbeck and Laposata, 1993). Alternatively, the effect may be mediated through inhibition of 15-HETE production (Smith *et al*, 1999), since we have shown 15-HETE to also increase NF-*κ*B nuclear binding and I*κ*B*α* degradation at the same concentrations that cause an increase in protein degradation (unpublished results). It is possible that 15-HETE could result in the generation of reactive oxygen species, which might regulate the redox-sensitive NF-*κ*B (Bonizzi *et al*, 1996; Li *et al*, 1998), possibly through oxidation of constituent proteins that augment, or promote the release from or degradation of I*κ*B.

Although the present study does not confirm a role for NF-*κ*B in ubiquitin–proteasome proteolysis, it does suggest that NF-*κ*B is activated prior to the induction of this pathway by PIF. Further studies are in progress to establish the role of NF-*κ*B in proteasome proteolysis.
